# Supplementary material for: Response to interferons and antibacterial innate immunity in the absence of tyrosine‐phosphorylated STAT1
Source: EMBO Rep. 2016 Feb 12;17(3):367–82. doi: 10.15252/embr.201540726 (PMC4772975; doi:10.15252/embr.201540726)
Supplement: Supplementary file 1 — Appendix [file EMBR-17-367-s001.pdf]

Appendix

Contents:

Appendix Table S1

Appendix Table S1: List of primers used for Q-PCR and ChIP experiments.

primer sequence	gene
for TCA GAA TGA GTG GTG GTT GC<br>rev GAC CTT TCA AAT GCA GTA GAT TCA	Ifnβ
for GCT GCC TAT GAT GTC TCG TTT<br>rev TGC TTT TCC GTA TGT TGT GCT	Stat1
for TCC TGC CAA TGG ACG TTC G<br>rev GTC CCA CTG GTT CAG TTG GT	Stat2
for CCG AAG ACC TTA TGA AGC TCT TTG<br>rev GCA AGT ATC CCT TGC CAT CG	Irf1
for ATT TCG GTC GTA GGG ATC TGG<br>rev GCA CAG CGG AAG TTG GTC T	Irf7
for CCT CAG GCA AAG TAC GCT G<br>rev GGG GTG TCC TAT GTC CCC A	Irf9
for GAC TAC CAC TGA GAT GAC CCA GC<br>rev ATT TCC TCC CCA AAT GTT TTC A	Mx1
for CCA GTT CCT CTC AGT CCC AAG ATT<br>rev TAC TGG ATG ATC AAG GGA ACG TGG	Mx2
for GAC CTT TCA AAT GCA GTA GAT TCA<br>rev CCA AAG GGG ATA GTG GGT GTC	CIITA
for ATG GCC TGG GAC CTA AAG<br>rev TTA GGC ACA CTG GTC CCC	Isg15
for GGA GAG CAA TCT GCG ACA G<br>rev GCT GCC TCA TTT AGA CCT CTG	Ifit2
for CCT CGC AGC CCT GGA GTG TT<br>rev TGC GTT GCC TCC CAA ACC CC	Ifit3
for ACT CCG TGA CTA CCT GAG TTC CTT<br>rev GCA TCT CAC CCT CCA CAA CCA CT	Sosc1
for CAT GGC CTT CCG TGT TCC TA<br>rev GCG GCA CGT CAG ATC CA	Gapdh
for GGG GTG TCC TAT GTC CCC A<br>rev GCA GCT GCC AGG GCT CAG AC	Mx2-prox ISRE
for GGT CGG GTG TAG TTT GAG GA<br>rev GCC AAG GTG GCT GTA GAT GT	Irf7 ISRE
