# Supplementary material for: Response to interferons and antibacterial innate immunity in the absence of tyrosine‐phosphorylated STAT1
Source: EMBO Rep. 2016 Feb 12;17(3):367–82. doi: 10.15252/embr.201540726 (PMC4772975; doi:10.15252/embr.201540726)
Supplement: Supplementary file 2 — Expanded View Figures PDF [file EMBR-17-367-s002.pdf]

Expanded View Figures

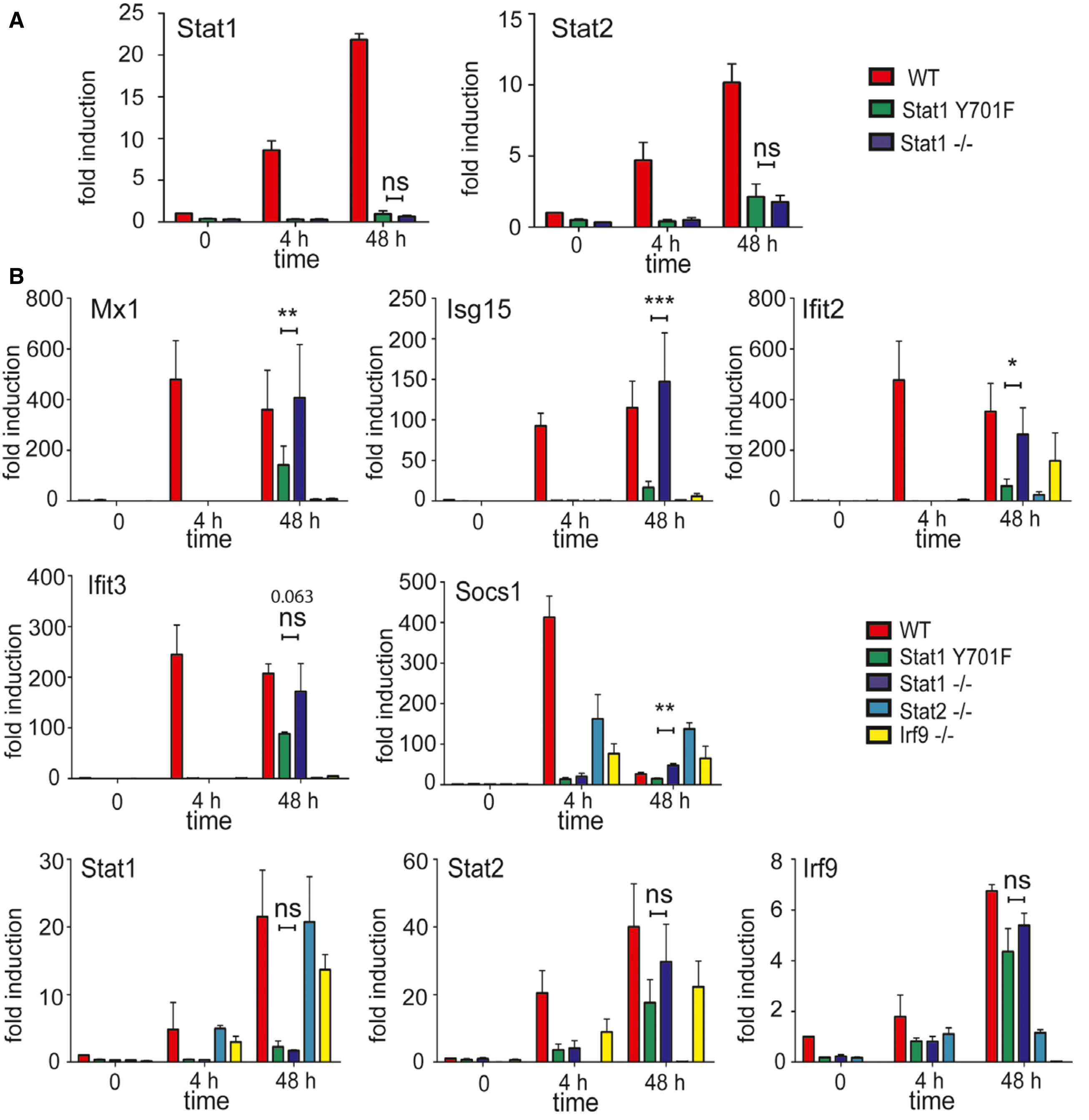

Figure EV1. STAT1Y701F inhibits late STAT1-independent, STAT2/IRF9-dependent ISG expression in response to IFNβ.

A Expression of Stat1 and Stat2 genes. Bone marrow-derived macrophages (BMDMs) of wild-type (WT), $Stat1^{Y701F}$, and $Stat1^{-/-}$ mice were treated with 5 ng/ml of IFNγ (IFNg) for 4 or 48 h. Gene expression was measured by qPCR and normalized to Gapdh and to the expression levels in uninduced wild-type cells.

B Expression of type I IFN-induced genes. BMDMs of wild-type (WT), $Stat1^{Y701F}$, $Stat1^{-/-}$, $Stat2^{-/-}$, and $Irf9^{-/-}$ mice were treated with 250 IU/ml of IFNβ for 4 or 48 h. Gene expression was measured by qPCR and normalized to Gapdh and to the expression levels in uninduced wild-type cells.

Data information: The bars in (A) and (B) represent a mean value of three independent experiments. Error bars represent standard error of the mean (SEM) and asterisks denote level of statistical significance (ns, $P > 0.05$; $^{*}P \leq 0.05$; $^{**}P \leq 0.01$; $^{***}P \leq 0.001$); the *P*-values were calculated using paired ratio *t*-test.

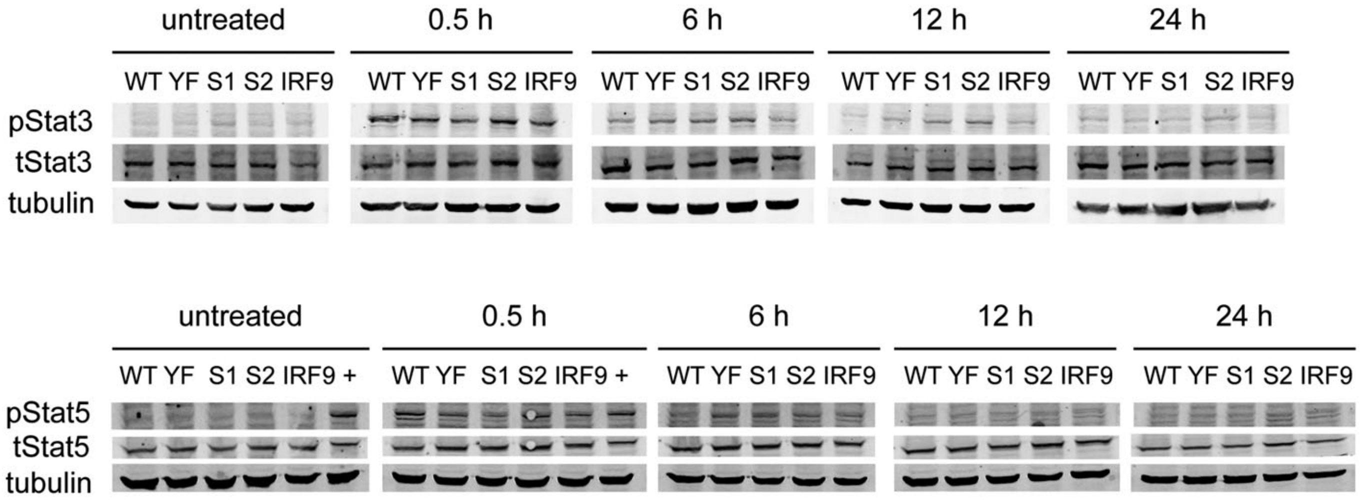

Figure EV2. Deletion of ISGF3 subunits or Stat1^{Y701F} mutation is without effect on the expression or IFNβ-stimulated phosphorylation of STAT3 and STAT5.
Western blot analysis of STAT3 and STAT5 tyrosine phosphorylation. Bone marrow-derived macrophages (BMDMs) of wild-type (WT), Stat1^{Y701F} (YF), Stat1$^{-/-}$ (S1), Stat2$^{-/-}$ (S2), and Irf9$^{-/-}$ (IRF9) mice were treated with 250 IU/ml of IFNβ for 0.5, 6, 12, or 24 h. Whole-cell extracts were collected and tested in Western blot for total STAT3 and STAT5 amounts and for their tyrosine phosphorylation at Y705 and Y694, respectively. The blots are representative of more than three independent experiments.

 © 2016 The Authors

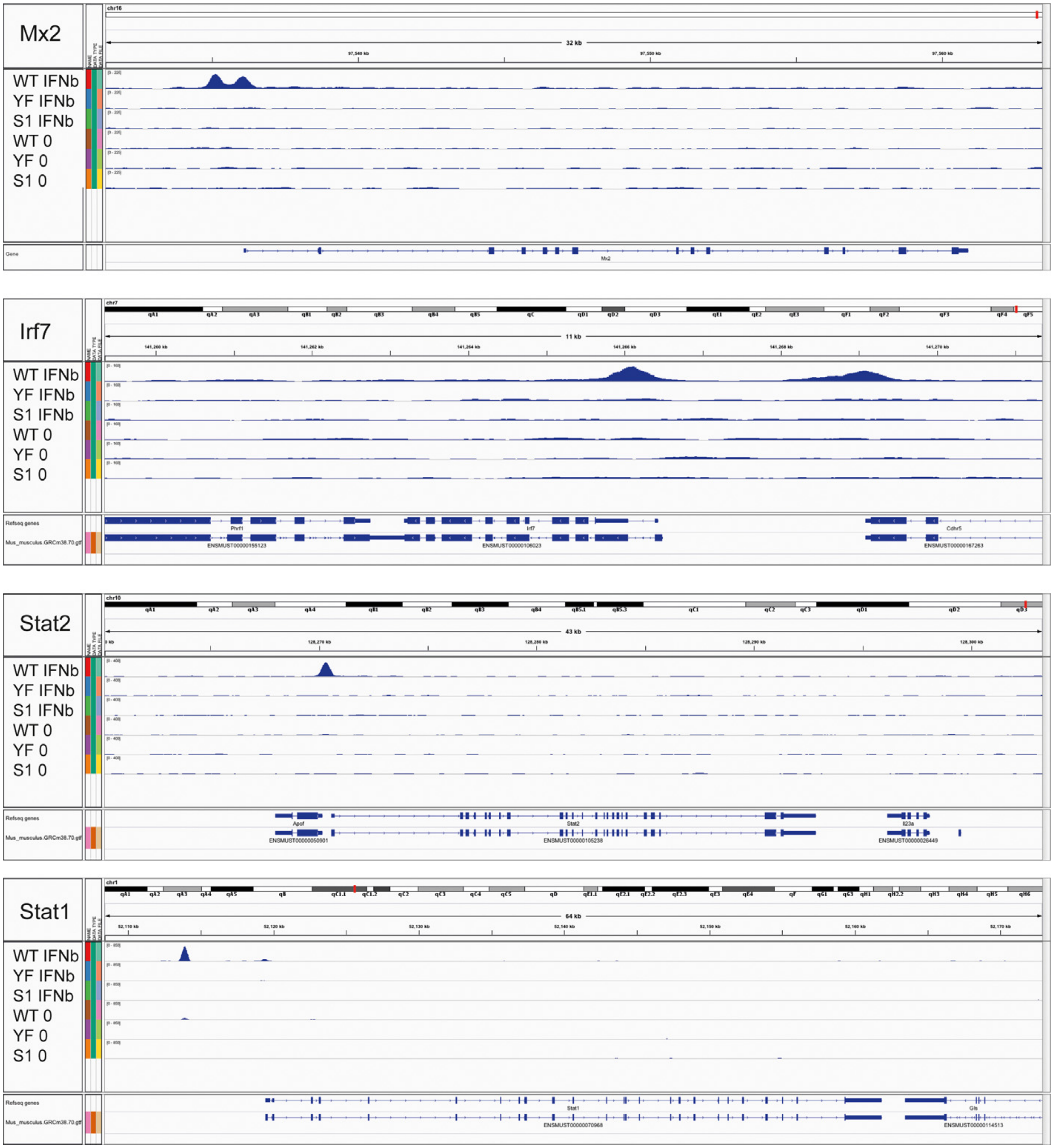

Figure EV3. STAT1Y701F mutant does not bind to ISRE sequences after IFNβ treatment.
Genomic analysis of STAT1 binding. ChIP-seq analysis was performed on bone marrow-derived macrophages (BMDMs) of wild-type (WT), $Stat1^{Y701F}$ (YF), and $Stat1^{-/-}$ (S1) mice. BMDMs were treated with 250 IU/ml of IFNβ (IFNb) for 2 h. Chromatin immunoprecipitation (ChIP) was performed using STAT1 antibody. Four genes are shown as representative examples. A genome-wide search did not produce evidence of STAT1Y701F binding to ISREs elsewhere in the genome. ChIP-Seq data for $Stat1^{Y701F}$ mutant and $Stat1^{-/-}$ samples are deposited at ArrayExpress under accession number E-MTAB-3597 (https://www.ebi.ac.uk/arrayexpress/experiments/E-MTAB-3597/) and for wild-type Stat1 sample are deposited at ArrayExpress under accession number E-MTAB-2972 (https://www.ebi.ac.uk/arrayexpress/experiments/E-MTAB-2972/).

© 2016 The Authors

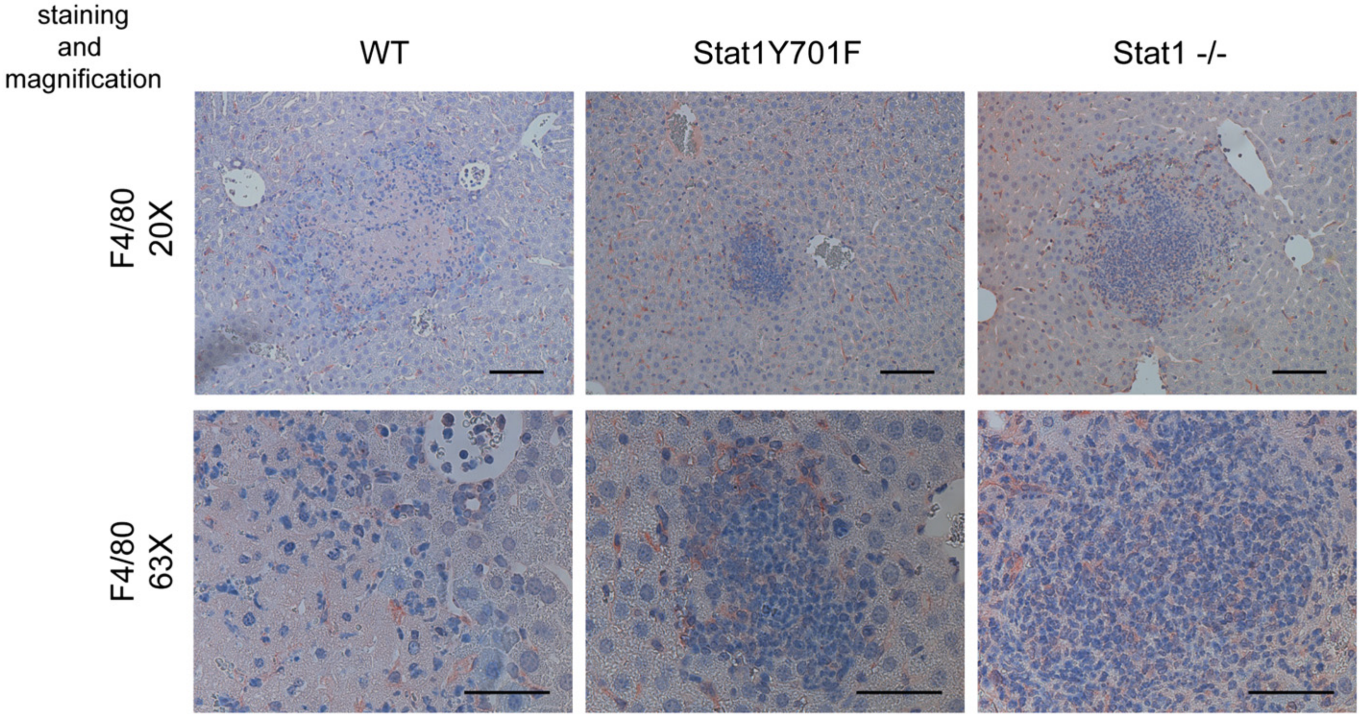

Figure EV4. Immune cell infiltrates in Stat1^{Y701F} mice livers are smaller in numbers and size compared to Stat1$^{-/-}$ livers, and are mostly composed of F4/80-negative cells.
Wild-type (WT), Stat1^{Y701F}, and Stat1$^{-/-}$ mice were infected by intraperitoneal injection of 1×10^2 *L. monocytogenes* for 48 h. Immunohistochemistry was performed on liver sections using F4/80-specific antibody. The scale bars on 20× magnification images represent 100 and 50 μm on the 63× magnification images.

 © 2016 The Authors
